# The impact of ocular trauma during the Nepal earthquake in 2015

**DOI:** 10.1186/s12886-017-0429-z

**Published:** 2017-03-28

**Authors:** E. Pradhan, B. Limbu, S. Thakali, N. S. Jain, R. Gurung, S. Ruit

**Affiliations:** 1 Tilganga Institute of Ophthalmology, Ring Road, Gaushala, Kathmandu, 44600 Nepal; 2 University of New South Wales, Sydney, Australia

**Keywords:** Earthquake, Ocular Trauma, Closed globe injury

## Abstract

**Background:**

Nepal was struck by a massive earthquake on the 25th April 2015 and major aftershock on the 12th of May 2015, resulting in widespread devastation with a death toll in the thousands. The burden of ocular trauma resulting from the recent earthquakes in Nepal has not been described thus far. The aim of this study was to determine the types of ocular injuries sustained in the earthquake in Nepal and its management in Tilganga Institute of Ophthalmology (TIO) in Gaushala, Kathmandu.

**Methods:**

This is a hospital-based retrospective study of patients presenting to TIO following repeated earthquake. Variables that were recorded included patients’ presenting symptoms and time to presentation, visual acuities at presentation and at follow-up, diagnosis of ocular injury and surgery performed.

**Results:**

There were 59 cases of earthquake victims visiting TIO, Gaushala, Kathmandu from April 2015 to July 2015, with 64 affected eyes due to 5 cases of bilateral involvement. The majority of patients were from the district Sindhupalchowk (14 cases, 23.7%), which was the epicenter of the main earthquake. The average duration between the earthquake and presentation was 13 · 9 days (range 1–120 days). Closed globe injury was most frequent (23 cases), followed by open globe injuries (8 cases). While 24 patients (38%) initially presented with a visual acuity <3/60 in their affected eye, 15 patients (23%) had a visual acuity of <3/60 on follow-up. A variety of surgical treatments were required including anterior and posterior segment repair.

**Conclusions:**

Immediate management of ocular trauma is critical in order to prevent blindness. Characterizing the burden of earthquake-related ocular trauma will facilitate planning for service provision in the event of a future earthquake in Nepal, or in countries, which are similarly at risk of having natural disasters.

**Electronic supplementary material:**

The online version of this article (doi:10.1186/s12886-017-0429-z) contains supplementary material, which is available to authorized users.

## Background

On the 25th of April, a 7.8 Richter earthquake struck Nepal with its epicenter 77 km from Kathmandu. A 7.3 Richter aftershock subsequently occurred on the 12th of May, equidistant from Kathmandu [1–3]. The earthquakes resulted in more than 8000 fatalities and 22,000 injuries all over the country [1]. National heritage sites in the heart of the city were destroyed and thousands were left homeless as entire villages were almost flattened. Tilganga Institute of Ophthalmology (TIO) was one of the eye hospitals to provide emergency services for earthquake-related ocular trauma patients.

Ocular trauma remains an important cause of visual impairment worldwide. Annually, over 2.5 million Americans suffer an eye injury, and more than half a million blinding injuries occur worldwide [4]. In Nepal, ocular trauma is widespread in comparison with more developed countries. Trauma-related ocular blindness was reported to affect 2.4% of the population according to the Nepal Blindness survey conducted in 1981 [5], while the prevalence of ocular trauma was reported to be 0.7% according to the more recent Bhaktapur eye study in 2001 [6, 7]. Corneal trauma leading to ulceration is the second most common cause of monocular blindness after cataract in Nepal. Major causes of ocular trauma in Nepal include agricultural and domestic work, road traffic accidents and physical assault [8]. Although there have been many reports describing ocular trauma in Nepal [6, 8–13], little is known about the burden of ocular trauma during the recent earthquakes in Nepal or burden due to other such natural disasters around the world.

Knowledge of the causes of eye injuries can aid in guiding preventative strategies and optimizing management capacity. For example, implementation of seat-belt legislation and application of occupational eye protection have significantly reduced the number of ocular injuries [14]. In the case of natural disasters however, trauma generally cannot be anticipated and the mode of ocular injury may vary widely according the scenario of the incident. Understanding the types and nature of ocular injuries sustained in natural disasters such as an earthquake will help to guide planning for ocular care for future natural disasters. This will be important particularly as a further major earthquake can occur in Nepal owing to its terrain [1]. The aim of this study was to determine the types of ocular injuries sustained in the earthquakes in Nepal and their subsequent management.

## Methods

This is a hospital-based retrospective study, including all patients presenting with ocular trauma to Tilganga Institute of Ophthalmology, a major tertiary eye hospital in Kathmandu, following the major earthquake of April 25th and aftershock on 12th May 2015. This study had ethical approval by Institutional Review Committee of TIO, which is a part of Nepal Health Research Council.

For each patient, a brief clinical history regarding the ocular injury and time of injury was obtained. Visual acuity was assessed using an internally illuminated standard Snellen’s chart. All the subjects underwent a detailed eye examination under a slit-lamp bio-microscope (Haag-Striet 900) with intraocular pressure measurement with Goldman’s applanation tonometry in required cases. Fundus evaluation was performed under full mydriasis with 90 Dioptre leans, or binocular indirect ophthalmoscope as appropriate. Relevant investigations such as an Ultrasound B scan, X-ray orbit and skull, Computerized Tomography scan and Magnetic Resonance Imaging were requested whenever indicated. An ophthalmologist examined all the patients and appropriate interventions, whether medical or surgical, were undertaken immediately. Data of each case including demographics, visual acuities and examination findings were recorded and analyzed (Additional file 1).

Most of the ocular injury were either due to falling objects or being trapped underneath, therefore the classification was adopted from the modified form of International Ocular Trauma Classification and Birmingham Eye Trauma Terminology [15, 16].

## Results

**Fig. 1:**
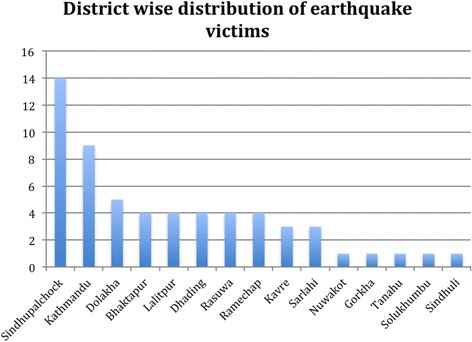
District wise distribution of patients during earthquake

There were 59 cases of earthquake victims who sought treatment at TIO from April 2015 to July 2015, with 64 affected eyes due to 5 cases of bilateral involvement. The average age of the patients presenting with ocular injury was 38 years (range 4 to 75 years). Among the total victims, 12 patients (20%) were children. Males were slightly more affected; 52% patients were male and 48% were female. The average duration between the earthquake and presentation was 13.9 days (range 1–120 days). (Table 1) The majority of patients were from the district Sindhupalchowk (*n* = 14, 23.7%) followed by Kathmandu (*n* = 9, 15.3%), among many other districts (Fig. 1).

**Table 1:** Demographic data and presentations of patients

Characteristic	Number	Percentage
Age		
Mean Age	38 years (4–75)	
Sex		
Male	34	52
Female	25	48
Laterality		
Unilateral	54	91.5
Bilateral	5	8.5
Presenting Complaint		
Blurring of vision	35	59
Red eye	15	25
Cut Injury	5	9
Diplopia	3	5
Foreign body	1	2
Average presentation duration	13.9 days (1–120)

Uniocular involvement occurred in 54 cases (91.5%), with the right and left eyes being equally involved, and binocular involvement occurred in 5 cases (8.5%) (Table 1). The chief ocular findings at the time of presentation were blurring of vision (35 cases), red eye (15 cases), cut injury (5 cases), diplopia (3 cases) and a foreign body (1 case) (Table 1). While 24 patients (38%) initially presented with a visual acuity <3/60 in their affected eye, at follow-up 15 patients (23%) had a visual acuity of <3/60 (Table 2). No patient developed bilateral blindness due to trauma. Vision in the eye unaffected by trauma was generally unimpaired, with only seven cases demonstrating low vision (11.6%) due to pre-existing eye conditions (Table 2).

**Table 2:** Visual acuity at presentation and follow up, and fellow eye vision among ocular trauma patients

Vision Category	Presenting BCVA	After treatment BCVA	Fellow eye vision
6/6–6/18	25 (39%)	36 (56.2%)*	52
6/18–6/60	12 (18.7%)	9 (14%)	5
6/60–3/60	3 (4.6%)	4 (6.2%)	1
<3/60–1/60	5 (7.8%)	3 (4.6%)	0
<1/60–PL•	16 (25%)	6 (9.3%)	1
NPL	3 (4.6%)	6 (9.3%)	0

• Perception of light

*In all cases of low vision (<6/18 according to WHO) in the unaffected eye, the cause was pre-existing conditions and not bilateral trauma

Closed globe injury was most frequent (23 cases), followed by open globe injuries (8 cases) and corneal ulcer (7 cases) (Table 3). Out of five bilateral cases, three had conjunctivitis, one had vitreous hemorrhage and one had iritis.

**Table 3:** Diagnosis of patients affected by earthquake

Diagnosis	Number of Victims	Percentage
Conjunctivitis	4	6.8
Lid Laceration	3	5.1
Lid Laceration with Canaliculus	1	1.7
Open Globe I	5	8.5
Open Globe II	3	5.1
Open Globe Zone III	1	1.7
Close Globe Zone I	9	15.3
Close Globe Zone III	14	23.7
Orbital bone Fracture	3	5.1
Corneal Ulcer + FB^a^ Corneal	7	11.9
Post traumatic optic atrophy	3	5.1
Intra ocular Foreign Body	4	6.8
Post Traumatic Glaucoma	1	1.7
Corneal Abrasion	1	1.7

^a^ *FB* foreign body

A similar proportion of patients were treated medically (*n* = 31, 52.5%) and surgically (*n* = 28, 47.5%). A variety of surgical treatments were required including anterior and posterior segment repair (Table 4).

**Table 4:** Different treatment modality for patients affected by earthquake

Surgery Performed	Number of Victims	Percentage
Corneal FB^a^ Removal	5	17.9
Corneal +/− Scleral Repair	8	28.6
Lid Repair	4	14.3
Orbital bone fracture repair	2	7.1
Evisceration	1	3.6
PPV^b^ + EL ^c^+/− SO^d^	7	25
PRP^e^	1	3.6

^a^Foreign Body

^b^Pars Plana Vitrectomy

^c^Endolaser

^d^Silicon Oil

^e^Panretinal photocoagulation

## Discussion

Although visual loss due to ocular trauma has been reported to be widespread in Nepal [6, 7, 9–13], the burden of ocular trauma resulting from the recent earthquakes in Nepal has not been described thus far. In this series of 59 patients seen at TIO, a variety of ocular injuries were managed. The mode of trauma was direct and indirect impact with closed and open globe injury. The primary mechanism for injury was reported by patients to be trauma from collapsing structures in buildings or falls from heights as patients escaped buildings. Many patients were from the Everest base camp, where an avalanche resulted in multiple casualties. Several patients underwent immediate surgery to save the integrity of the eye as well as the vision.

The management of ocular injuries at TIO was complicated by the chaos associated with a large-scale natural disaster. Due to the numerous minor aftershocks and fear of a repeat earthquake, clinicians often felt unsafe entering the hospital building, preventing some injured patients from receiving prompt management. In the event of a future earthquake, the authors recommend preparing a separate, temporary tent shelter to provide an emergency service, in order to mitigate concerns about the safety of the building and allow more patients to benefit from early surgery. A triaging system in the casualty area would also be useful to ensure serious injuries are prioritized and will be outlined in a new policy at TIO.

Despite appropriate management, a quarter of patients were observed to have monocular blindness (visual acuity <3/60) in their affected eye at follow-up, however no patient developed bilateral blindness. In contrast, previous series in Nepal have demonstrated monocular blindness in only 5–10% of patients on follow-up [8], even when presentation was delayed [13], reflecting the greater severity of injuries in the current series. Blindness may be devastating for an individual, with loss of vision in one or both eyes being classified as a 24% or 85% whole person impairment respectively [17]. Severe ocular trauma may further impose significant direct costs due to the need for specialist medical care, hospitalization, follow-up appointments and visual rehabilitation, as well as indirect costs due to loss of income and time away from caregiving roles [18, 19]. The social cost due to lost productivity may be substantial, particularly as most individuals affected by ocular trauma are young [14].

Natural disasters such as earthquakes can pose significant challenges for countries’ health systems. Mass casualties often occur and management of patients may be complicated by concurrent damage to hospitals and transport infrastructure which can prevent patients from being able to access healthcare [1–3]. These issues are exacerbated in a developing country such as Nepal, where poorly-build houses led to numerous casualties and health services prior to the earthquake were already strained in servicing the demands of the population [3]. The mountainous terrain further complicated transport and search and rescue operations, contributing to a delay in emergency response before international teams arrived. While the primary burden of earthquake-related trauma was orthopaedic [20, 21], all medical fields were involved in managing patients in the aftermath of the earthquake [22–24]. Ophthalmology services in the acute setting following a natural disaster are particularly important, as even minor ocular injuries that are sustained may become sight threatening if not managed promptly.

The spectrum of ocular trauma resulting from the earthquake contrasted markedly with that of general ocular trauma cases seen in Nepal due to agriculture or domestic work [6, 8–13]. The equal male:female ratio contrasts with studies examining general trauma cases in Nepal, which report a predominance of eye injuries in males [6, 8–13]. This ratio has been proposed to reflect that males are often exposed to a higher risk of ocular injury, however may also reflect an apparent lower incidence in women due to gender-related barriers in accessing care [9]. A much higher proportion of cases also presented with very low visual acuity (40% of cases <3/60) compared with comparable studies of general ocular trauma cases (2.7% of cases <3/60 in reported a series presenting to Dhulikhel hospital [9]. This was not surprising as TIO is the major tertiary referral centre for Ophthalmology in the area and would be expected to be referred cases on the more severe end of the spectrum. Indeed, the most common injury in this study was closed globe injuries, in contrast to previous series conducted in Nepal in which less serious injuries such as corneal abrasions were predominant [6]. The mean age and age range was comparable to previous series of ocular trauma in Nepal [6, 8–13].

A further explanation for the low initial visual acuity on presentation might be delays in healthcare presentation, as the average duration before presentation was a fortnight. In contrast, 50–70% of patients presented within 1 day of injury in comparable series of ocular trauma patients in Nepal [9, 13]. Late presentations could have occurred as patients first presented to closer institutions which could not provide specialized eye care, with other serious injuries such as orthopedic injuries which had to be managed first [20]. Another reason could be a lack of awareness or education about the potentially sight-threatening nature of eye injuries. Such a lack of knowledge has been reflected in previous population wide studies and has been associated with worse visual outcomes [6, 9]. Further in rural areas, a lack of transport and availability of eye care facilities mean many patients consult traditional healers or medical shopkeepers before presenting at hospital [9, 11], further delaying presentation.

There have been a few other reports on the management of ocular-related conditions in other natural disasters, which are of interest for comparison. Anecdotal reports following the major earthquake in Haiti reported frequent open globe injuries, crush injuries, ptosis and head injuries associated with cranial nerve defects [25]. However, in our study, closed globe injury was found most frequently, followed by open globe injury and corneal ulcer. Reports describing outreach Ophthalmological management in the 1 month following the Great Japan earthquake also described longer term management following natural disasters. Key issues that were addressed included replacing lost eye glasses, managing pre-existing ocular diseases that required medical therapy and providing surgical follow-up after the acute management of ocular trauma [26–28].

## Conclusions

Immediate management of ocular trauma is critical in order to prevent blindness. In this study, delayed presentations were frequent and up to a quarter of patients had sustained severe vision loss at follow-up. The management of earthquake-related trauma is compounded by difficulties in patients accessing specialist healthcare, multiple other injuries and damage to transportation and hospital infrastructure. The institution of a triaging system in the casualty area might help to prioritise injuries requiring urgent attention in the event of a future mass casualty event. Characterizing the burden of earthquake-related ocular trauma will facilitate planning for service provision in the event of a future earthquake in Nepal, or in countries, which are similarly at risk of having natural disasters.

## Additional file

Additional file 1: Is the Raw Data of Earthquake victims treated at Tilganga Institute of Ophthalmology in Kathmandu in 2015. The file includes two sheets, the Data sheet and Coding sheet. The Data Sheet includes data of each patient in terms of age, sex, address, the timing of trauma and the timing of treatment in the hospital, as well as the vision of the affected eye, laterality, management and the final vision. The Coding sheet covers the code for each parameter. The data of personal identity information will not be made available in order to protect the patients’ privacy. (XLS 38 kb)
